# UHPLC-MS profiles and antidiarrheal activity of *Quercus coccinea* münchh. and *Quercus robur* L. employing *in vivo* technique

**DOI:** 10.3389/fphar.2023.1120146

**Published:** 2023-02-17

**Authors:** Mohamed S. Mady, Reham R. Ibrahim, Elsayed K. El-Sayed, Mohamed El-Shazly, Lo-Yun Chen, Kuei-Hung Lai, Fatheya S. El Shaarawy, Fatma A. Moharram

**Affiliations:** ^1^ Faculty of Pharmacy, Pharmacognosy Department, Helwan University, Cairo, Egypt; ^2^ Faculty of Pharmacy, Pharmacology and Toxicology Department, Helwan University, Cairo, Egypt; ^3^ Faculty of Pharmacy, Pharmacognosy Department, Ain-Shams University, Cairo, Egypt; ^4^ College of Pharmacy, Graduate Institute of Pharmacognosy, Taipei Medical University, Taipei, Taiwan; ^5^ College of Pharmacy, Ph.D Program in Clinical Drug Development of Herbal Medicine, Taipei Medical University, Taipei, Taiwan; ^6^ Taipei Medical University Hospital, Traditional Herbal Medicine Research Center, Taipei, Taiwan

**Keywords:** antidiarrheal, polyphenolic compounds, *Quercus coccinea*, *Quercus rubur*, UHPLC-MS

## Abstract

**Introduction:** *Quercus* L. genus (Oak) belongs to the family Fagaceae and their galls are used commercially in leather tanning, dyeing, and ink preparation. Several *Quercus* species were traditionally used to manage wound healing, acute diarrhea, hemorrhoid, and inflammatory diseases. The present study aims to investigate the phenolic content of the 80% aqueous methanol extract (AME) of *Q. coccinea* and *Q. robur* leaves as well as to assess their anti-diarrheal activity.

**Methods:** Polyphenolic content of *Q. coccinea* and *Q. robur* AME were investigated using UHPLC/MS. The antidiarrheal potential of the obtained extracts was evaluated by conducting a castor oil-induced diarrhea *in-vivo* model.

**Result and Discussion:** Twenty-five and twenty-six polyphenolic compounds were tentatively identified in *Q. coccinea* and *Q. robur* AME, respectively. The identified compounds are related to quercetin, kaempferol, isorhamnetin, and apigenin glycosides and their aglycones. In addition, hydrolyzable tannins, phenolic acid, phenyl propanoides derivatives, and cucurbitacin F were also identified in both species AME of *Q. coccinea* (250, 500, and 1000 mg/kg) exhibited a significant prolongation in the onset of diarrhea by 17.7 %, 42.6%, and 79.7% respectively while AME of *Q. robur* at the same doses significantly prolonged the onset of diarrhea by 38.6%, 77.3%, and 2.4 folds respectively as compared to the control. Moreover, the percentage of diarrheal inhibition of *Q. coccinea* was 23.8%, 28.57%, and 42,86% respectively, and for *Q. robur* 33.34%, 47.3%, and 57.14% respectively as compared to the control group. Both extracts significantly decreased the volume of intestinal fluid by 27%, 39.78%, and 50.1% for *Q. coccinea* respectively; and by 38.71%, 51.19%, and 60% for *Q. robur* respectively as compared to the control group. In addition, AME of *Q. coccinea* exhibited a peristaltic index of 53.48, 47.18, and 42.28 with significant inhibition of gastrointestinal transit by 18.98%, 28.53%, and 35.95 % respectively; while AME of *Q. robur* exhibited a peristaltic index of 47.71, 37, and 26.41 with significant inhibition of gastrointestinal transit by 27.72%, 43.89%, and 59.99% respectively as compared with the control group. Notably, *Q. robur* showed a better antidiarrheal effect in comparison with *Q. coccinea* and, the highest effect was observed for *Q. robur* at 1000 mg/kg as it was nonsignificant from the loperamide standard group in all measured parameters.

## Graphical Abstract

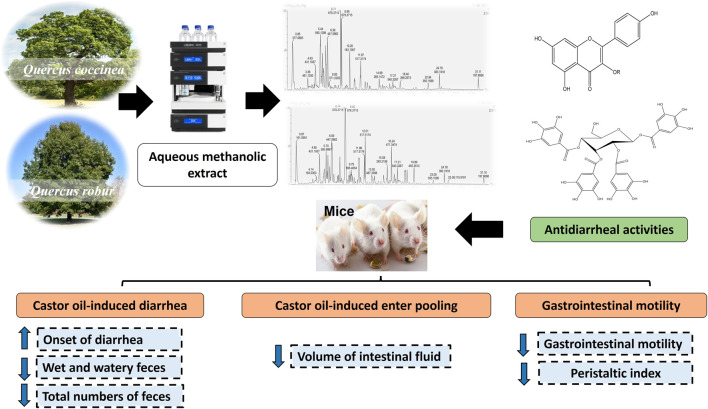

## 1 Introduction

Diarrhea is commonly well-defined as an unusual passage of the unformed stools or liquid accompanied by increasing defecation rate, and abdominal discomfort (Guerrant et al., 2001). Diarrhea can be categorized into acute, persistent, and chronic based on the duration of the symptoms. Acute diarrhea lasts less than 2 weeks and is caused by a bacterial and viral infection or by some medications. It can be resolved quickly while the persistent one starts suddenly and lasts from two to four weeks. On the other hand, chronic diarrhea lasts more than four weeks. Chronic and persistent diarrhea is commonly due to a functional disorder (Wingate et al., 2001; Thapar and Sanderson, 2004). Although diarrhea can be prevented and treated, it still represents one of the most serious health problems in the world, particularly in children under five years of age. It is a more challenging condition in developing countries. It is considered the second leading cause of mortality of death among children globally (Jaradat et al., 2016). The treatment of diarrhea usually focuses on reducing the pain associated with bowel movements. However, some of the available treatments are ineffective, unsafe, antagonize the effect of each other (Sinan et al., 2020), and/or promote resistance (Sinan et al., 2021). Most people in developing countries depend on folk medicine for the treatment of different diseases including diarrhea (Park, 2021). Medicinal plants are culturally accepted, available, and inexpensive in comparison to modern medicine. Several reports reported the use of medicinal plants as antidiarrheal agents since they can stimulate water absorption and decrease electrolyte secretion and intestinal motility (Agbor et al., 2004; Behera et al., 2006; Oben et al., 2006). They can also stimulate antispasmodic properties (Lozoya et al., 2002). The potency of medicinal plants as antidiarrheal agents depends on their content of secondary metabolites. It was reported that medicinal plants containing tannins, flavonoids, saponins, alkaloids, steroids, and terpenoids exhibited antidiarrheal activity (Chitme et al., 2004; Yadav and Tangpu, 2007; Brijesh et al., 2009). Flavonoids can inhibit intestinal motility and hydro electrolytic secretions (Venkatesan et al., 2005). Tannins form protein tannates through denaturation of the intestinal mucosa proteins. This effect can decrease the secretion and intracellular Ca^2+^ inner current or may activate the calcium pumping system encouraging muscle relaxation (Belemtougri et al., 2006). *Quercus* L. genus (family Fagaceae) is commonly known as oak. It comprises about 450 species of deciduous or evergreen trees widely spread in North Africa, Asia, Europe, and North, Central, and South America (Burlacu et al., 2020). The barks, woods, and galls of *Quercus* species have been commercially used since ancient times due to their tannins content. Oak galls were used in leather tanning, dyeing, and ink preparation (Şöhretoğlu and Renda, 2020). *Quercus* species were traditionally used for several disorders such as antimicrobial, wound healing, acute diarrhea, hemorrhoids, and inflammatory diseases (Şöhretoğlu and Renda, 2020). The anti-inflammatory, antibacterial, hepatoprotective, antidiabetic, gastroprotective, and antioxidant activity were assessed in previous reports (Moharram et al., 2015; Taib et al., 2020). It was reported that the biological effects of *Quercus* species were attributed to their high content of polyphenolic compounds which possess anti-inflammatory, antioxidant, and antidiarrheal activities (Salem et al., 2016; Dróżdż and Pyrzynska, 2018; Vong et al., 2018). The identified polyphenolic compounds in *Quercus* species were related to flavonoid, tannins and proanthocyanidins classes (Şöhretoğlu and Renda, 2020).

*Q. robur* L., commonly known as the English or pedunculate oak, is a large, lobed, deciduous, and broadleaved tree, which is native to Europe (Eaton et al., 2016). It was traditionally used for sore throats, anal fissures, hemorrhoids, and skin problems. Its decoction is used for diarrhea treatment, rectal bleeding, and dysentery (Farag et al., 1998). The amount of hydrolysable and condensed tannins in *Q. robur* and other *Quercus* species is seasonally dependent. It was reported that the highest insect attack on oak leaves occurs in early spring, which links to the time when the amount of hydrolysable tannins is minimum and condensed tannins are almost free (Scalbert and Haslam, 1987; Moharram et al., 2015; Formato et al., 2022) Previous reports revealed that hydrolyzable tannins belonging to hexahydroxydiphenoylesters nucleus were identified from *Q. robur* leaves, bark, and wood (Scalbert et al., 1988; Herve du Penhoat et al., 1991; Vivas et al., 1995). Moreover, catechin derivatives were detected from its bark (Vovk et al., 2003). The UHPLC-QTOF-MS profiling for *Q. robur* leaves (Moharram et al., 2015; Unuofin and Lebelo, 2021; Formato et al., 2022) and stem bark (Unuofin and Lebelo, 2021) indicated the presence of phenolic acid, flavonoids, and triterpenoids. Moreover, isolation of hydrolysable tannins and flavonoids from *Q*. *robur* leaves were reported by Moharram et al. (2015), Unuofin and Lebelo (2021), Formato et al. (2022).

*Q. coccinea* Münchh (Scarlet oak, red oak, or Spanish oak) is a deciduous tree with broadleaf. Its common name comes from the color of its leaves in autumn. It is native to the eastern and central parts of the United States, but it is also widely distributed around the world (Burns and Honkala, 1990; Yang et al., 2019). In general, there is little information about the chemistry of *Q. coccinea* but like other oak species, it produces galls that are rich in phenolic compounds (Grieve, 1984). The present study aimed to tentatively identify the polyphenolic compounds in *Q. coccinea* and *Q. robur* leaves using UHPLC -MS. We also investigated the antidiarrheal activity of both species against experimentally induced diarrhea.

## 2 Material and methods

### 2.1 General experimental material

Solvents used for polyphenols extraction were supplied from El Nasr Pharmaceutical Chemicals Co., Egypt) while that for HPLC (Merck, Darmstadt, Germany), loperamide (Sigma- Aldrich, MO, United States), castor oil and charcoal were purchased from a general market.

### 2.2 Plant material

*Q. coccinea* Münchh. and *Q. robur* L. leaves were collected from the International Garden, Cairo, Egypt in November 2021. They were kindly identified by Dr. Therese Labib, a plant taxonomist at Mazhar Botanical Garden, Giza, Egypt. A voucher samples are 34 Qro/2022 and 34 Qro/222 for *Q. coccinea and Q, robur* respectively, were kept at the Herbarium of the Pharmacognosy Department Faculty of Pharmacy, Helwan University, Cairo, Egypt.

### 2.3 Sample preparation and extraction of phenolic compounds

*Q. coccinea* and *Q. robur* air-dried leaves (600 g) were extracted with 80% aqueous methanol under reflux (4 × 3 L) followed by filtration and evaporation under *vacuum* to yield 80 g and 100 g extracts of *Q. coccinea and Q. robur*, respectively. The residue was defatted with petroleum ether followed by evaporation of the solvent to yield 60 g and 85 g methanol-soluble portions of *Q. coccinea* and *Q. robur*, respectively. The samples were stored at −15°C until UHPLC/MS analysis.

### 2.4 UHPLC-MS separation and identification

UHPLC-MS/MS studies were achieved using the Waters SYNAPT G2 LC/Q-TOF (Waters Corporation, Milford, MA, United States) system. The separation of the target compounds was implemented on the column of RP C_18_ Waters Acquity UPLC BEH (Waters, 1.7 μm, 2.1 mm × 100 mm). The mobile phase used was a mixture of solvent (A) acetonitrile and solvent B) 0.1% formic acid/water with a flow rate of 0.4 mL/min. The gradient sequence was 0–1 min, 5% A; 1–16 min, 5%–99.5% A; 16–26 min, 99.5% A; 26–26.1 min, 99.5%–5% A; 26.1–28 min, 5% A. The column temperature was sustained at 40°C. All solvents used were filtered by a 0.45 μm membrane filter as well as the extract (5 mg) was dissolved in methanol (1 mL) at 5,000 ppm. The non-targeted MS1 and MS2 data were composed at the range of *m/z* 100–2,000. The automated data-dependent acquisition (DDA) attitude was applied in the MS2 scans, and the non-targeted selections of 5 precursor ions were fragmented with ramping of the collision energy from 10 to 50 eV. The attained MS records were confirmed by Waters Mass Fragment software (MassLynx4.1, Waters, MA, United States).

### 2.5 Animals

Swiss albino male mice at age of 5–7 weeks (20–25 g) were provided by the Biological Products and Vaccines breeding unit, Helwan, Egypt. Mice were kept in the standard condition and were free to access standard pelleted diet and water. All *in vivo* experiments were permitted by the scientific research ethics committee (Faculty of Pharmacy, Helwan University, no: 13A2022). The experiments were performed according to the European Community Directive (86/609/EEC), and the NIH Guidelines for the Care and Use of Laboratory Animals (8th edition).

### 2.6 Determination of median lethal dose (LD_50_)

For the determination of the LD_50_, mice (25 g) received orally 80% aqueous methanol extract (AME) dissolved in Tween 80 and distilled water with the aid of tween 80 with increasing doses till 5 g/kg. At the same time, the control group mice were administrated Tween 80 and distilled water. Mice’s general behavior and mortality percentage were noticed over 24 h (Mady et al., 2022).

### 2.7 *In vivo* anti-diarrheal activity

#### 2.7.1 Castor oil-induced diarrhea in mice

Castor oil-induced diarrhea in mice was performed according to Zhao et al. (2018). Forty-eight mice were fasted for 18 h and then classified into eight groups (n = 6). Group I received orally the vehicle and was considered an untreated control, group II taking loperamide orally (5 mg/kg) and served as the standard group. The mice in groups III-VIII received the AME of *Q. coccinea* and *Q. robur* orally using gavage and the concentrations were 250, 500, and 1,000 mg/kg b. wt. After one hour from oral administration of castor oil (0.2 mL) to each mouse and they were kept individually in a plastic cage, where its floor was covered with white blotting paper. For each mouse, the onset of diarrhea was recorded, and the numbers of normal, wet, and watery feces were calculated for 4 h.

$$Percentage\;of\;inhibition=\frac{Mean\;number\;of\;WFC-Mean\;number\;of\;WFT}{Mean\;number\;of\;WFC}X100$$

Where WFC and WFT were the wet Feces for the control and test groups, respectively.

#### 2.7.2 Castor oil-induced enter pooling in mice

The mice were divided as in the previous method. After one hour of the administration of AME and standard, each mouse received castor oil (0.5 mL) orally then 1 h later, mice were sacrificed using cervical dislocation and their abdomens were opened to isolate small intestines after ligation from the pylorus to the caecum. The intraluminal fluid was squeezed and placed in a graduated tube to determine the volume (Abdela, 2019).

#### 2.7.3 Castor oil triggered charcoal meal transit test

Fasted forty-eight mice were categorized into eight groups as previously described (Mekonnen et al., 2018). After one hour of the administration of AME and loperamide, each mouse in all groups was given castor oil (0.5 mL). One hour later, each mouse received 5% charcoal 0.5 mL orally suspended in distilled water. Thirty minutes later, all mice were sacrificed to isolate their small intestines (Mekonnen et al., 2018). The intestinal length traveled by the charcoal was recorded and represented as a percentage of the total intestine length (peristaltic index) (Mady et al., 2022).

$$Peristalsis\;index=\frac{Distance\;traveled\;by\;charcoal\;meal}{Length\;of\;small\;intestine}X100$$

$$\%\;inhibition=\frac{Mean\;Peristalsis\;index\;of\;control-Mean\;Peristalsis\;index\;of\;test}{Mean\;Peristalsis\;index\;of\;control}X100$$

#### 2.7.4 Statistical analysis

The results were stated as mean value ±SEM. Statistical analysis was done using Graph Pad Prism, version 8 (GraphPad Software Inc., United States), by one-way analysis of variance, followed by a Tukey’s test to measure the statistical significance between various groups. The value *p* < 0.05 was considered as significant.

## 3 Results

### 3.1 Identification of *Q. robur* and *Q. coccinea* polyphenols

**FIGURE 1:**
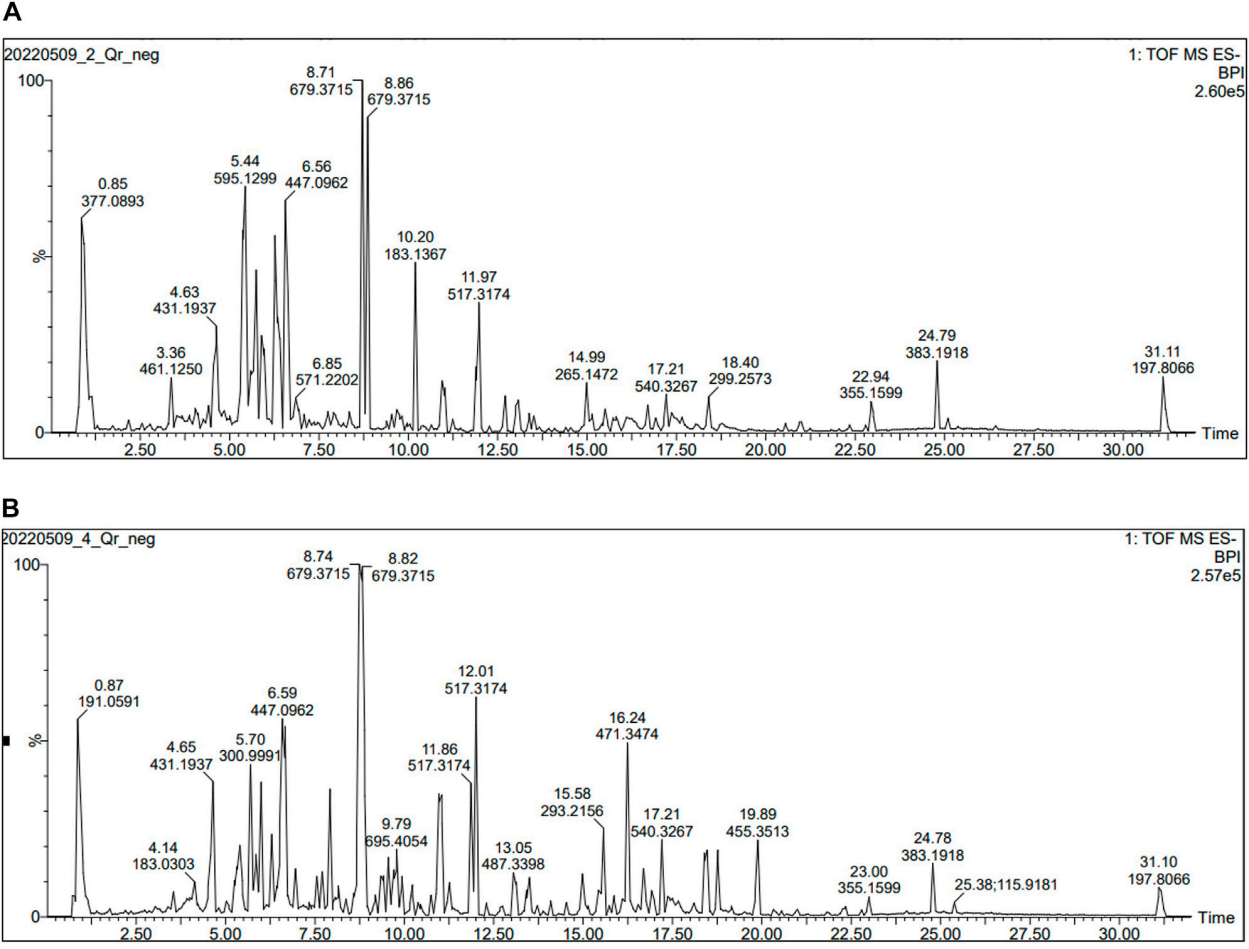
UHPLC-ESI-TOF MS profile (Total ion chromatogram acquired in negative ion mode) for **(A)** AME of *Q. robur* **(B)** AME of *Q. coccinea* extract.

UHPLC-MS analysis for the AME of both *Q. coccinea* and *Q. robur* leaves led to the tentative identification of twenty-five and twenty-six compounds in *Q. coccinea* and *Q. robur*, respectively belonging to phenolic acids, flavone, and flavonol aglycone and glycosides in addition to few tannins’ compounds (Table 1 and Figure 1). They were identified based on their molecular ion peak, fragmentation pattern, and comparison with the previously published data. The major compounds present in both *Quercus* species were further subjected to MS/MS to establish their structure (Table 2). Quercetin-*O* pentoside, quercetin pentose hexoside, rutin, isoquercitrin, and quercetin represented the quercetin derivatives. Kaempferol glucouronopyranoside, afzelin, kaempferol glucogallate, kaempferol hexoside, kaempferol pentoside, kaempferol diacetyl-*p*-coumaroylrhamnoside, tribuloside, and kaempferol represented the main kaempferol derivatives. Isorhamnetin*-O-* hexoside and isorhamnetin*-O-* rhamnoside were examples of isorhamnetin compounds. Apigenin*-O-*hexoside, methoxy apigenin-*O*-pentoside, vitexin, and isovitexin were examples of apigenin derivatives. Quinic, syringic, gallic, and ellagic acids represented the phenolic acids while ethyl and methyl gallate, and dimethyl ellagic acid-*O*-pentoside were examples of phenolic acid derivatives, caffeic acid hexoside, ferulic acid, and ferulic acid tri glucosides were examples of phenylpropanoids. Strictinin or its isomer and tetra galloyl glucose are related to hydrolysable tannins. Cucurbitacin F, an example of triterpene, was observed in both species. Isoquercitrin and isorhamnetin hexoside represented the major compounds in *Q. coccinea* while tribuloside and caffeic acid hexoside were the major components in *Q. robur*. Methoxy apigenin-*O*-pentoside, quercetin pentose hexoside, ellagic acid, kaempferol hexoside, and ferulic acid triglucoside were the major compounds detected in *Q. coccinea* and *Q. robur* leaves AME. Our study revealed that UHPLC MS/MS analysis of *Q. robur* leaves AME was consistent with the previously reported data (Moharram et al., 2015; Unuofin and Lebelo, 2021; Formato et al., 2022) which revealed that the identified flavonoids based on kaempferol and quercetin nucleus while our result demonstrated in addition to this compounds the presence of some tannins and flavonoids compounds related to apigenin and luteolin moiety. Moreover, Moharram et al. (2015) revealed the isolation of compounds **2**, **27**, **31** and **17** which identified also in our result. This difference in the results could be attributed to alterations in cultivation conditions, climate changes, and geographical origin of *Quercus* species (Holopainen et al., 2018; Stefi et al., 2022), while the components of *Q. coccinea* were identified for the first time.

**TABLE 1:** UHPLC profile tentative secondary metabolites identification for *Q. coccinea* and *Q. robur* leaves 80% aqueous methanol extract.

No	Rt (min)	Exact mass	[M-H]^ˉ^	MF	Name of compound	Species		References
						*Q. c*	*Q. r*	
**1**	0.87	192.06	191.0591	C_7_H_12_O_6_	Quinic acid	+		Nishimura et al. (1984), Sardans et al. (2014)
**2**	1.76	170.02	169.0149	C_7_H_6_O_5_	Gallic acid	+	+	Rahman et al. (2015)
**3**	1.18	342.10	341.1069	C_15_H_18_O_9_	Caffeic acid hexoside		+	Pacifico et al. (2019)
**4**	2.27	462.08	461.1343	C_21_H_18_O_12_	Kaempferol hexoside		+	Mahgoub et al. (2021)
**5**	3.11	462.08	461.125	C_21_H_18_O_12_	Di methyl ellagic acid *O* pentoside		+	Gao et al. (2021)
**6**	4.29	194.06	193.0522	C_10_H_10_O_4_	Ferulic acid	+	+	Aung et al. (2020)
**7**	4.55	432.11	431. 1937	C_21_H_20_O_10_	Apigenin-*O*-hexoside	+	+	Deveoglu et al. (2012)
**8**	4.63	432.11	431.1849	C_21_H_20_O_10_	Methoxy apigenin-*C*-pentoside	+	+	
**9**	4.66	432.11	431.1483	C_21_H_20_O_10_	Afzelin	+	+	Xu et al. (2018)
**10**	4.89	432.11	431.1574	C_21_H_20_O_10_	Vitexin	+	+	Elsayed et al. (2022)
**11**	4.89	432.11	431.1574	C_21_H_20_O_10_	Isovitexin	+	+	
**12**	5.22	434.08	433.0383	C_20_H_18_O_11_	Quercetin-*O*-pentoside	+		Sakar et al. (2005)
**13**	5.44	596.14	595.1299	C_26_H_28_O_16_	Quercetin-*O-*pentose hexoside	+	+	Unuofin and Lebelo (2021)
**14**	5.73	610.15	609.1497	C_27_H_30_O_16_	Rutin		+	Aung et al. (2020)
**15**	5.91	302.04	301.0370	C_15_H_10_O_7_	Quercetin	+	+	
**16**	5.99	464.10	463.0890	C_21_H_20_O_12_	Isoquercitrin	+		
**17**	6.08	302.01	300.9991	C_14_H_6_O_8_	Ellagic acid	+	+	Unuofin and Lebelo (2021)
**18**	5.44	594.16	593.1479	C_30_H_26_O_13_	Tribuloside	+		Unuofin and Lebelo (2021)
**19**	6.30	198.05	197.0818	C_9_H_10_O_5_	Ethyl gallate	+	+	Ou et al. (2015)
**20**	6.35	600.11	599.1036	C_28_H_24_O_15_	Kaempferol hexose gallate		+	Mahgoub et al. (2021)
**21**	6.59	448.10	447.0962	C_21_H_20_O_11_	Kaempferol glucouronopyranoside	+	+	Meng et al. (2001)
**22**	6.67	478.11	477.1068	C_22_H_22_O_12_	Isorhamnetin-*O*-hexoside	+		Romussi et al. (1988), Jiang et al. (2017), Formato et al. (2022), Stefi et al. (2022)
**23**	7.59	418.09	417.1580	C_20_H_18_O_10_	Kaempferol pentoside		+	Saunier et al. (2022)
**24**	8.55	462.12	461.1156	C_22_H_22_O_11_	Isorhamnetin-*O*-rhamnoside		+	Arámbula-Salazar et al. (2015)
**25**	8.72	680.22	679.3714	C_28_H_40_O_19_	Ferulic acid triglucoside	+	+	Usui et al. (2017)
**26**	9.55	286.05	285.0393	C_15_H_10_O_6_	Kaempferol	+	+	Bursal and Boğa (2018)
**27**	10.21	184.04	183.1367	C_8_H_8_O_5_	Methyl gallate	+	+	Ou et al. (2015)
**28**	11.97	518.32	517.3173	C_30_H_46_O_7_	Cucurbitacin F	+	+	Unuofin and Lebelo (2021)
**29**	13.12	824.20	823.1816	C_34_H_30_O_14_	Kaempferol diacetyl-*p*-coumaroylrhamnoside	+		Unuofin and Lebelo (2021), Formato et al. (2022)
**30**	13.48	198.05	197.1186	C_9_H_10_O_5_	Syringic acid	+	+	Dar Ms Fau - Ikram and Ikram (1979)
**31**	15.76	634.08	633.3814	C_27_H_22_O_18_	Strictinin or its isomer	+	+	Nishimura et al. (1986), Moharram et al. (2015)
**32**	21.056	788.11	787.3824	C_34_H_28_O_22_	Tetra galloyl glucose	+	+	Amr et al. (2021)

**TABLE 2:** MS/MS fragmentation for the major compounds present in *Q. coccinea* and *Q. robur* leaves 80% aqueous methanol extract.

Rt (min)	Exact mass	[M- H] ^ˉ^	Daughter fragment	Name of compound	Species	
					*Q. c*	*Q. r*
1.18	342.10	341.1069	179.05	Caffeic acid hexoside		+
4.63	432.11	431.1849	299.99	Methoxy apigenin-*O*-hexoside	+	+
5.44	596.14	595.1299	301.02	Quercetin *O-*pentose hexoside	+	+
5.99	464.10	463.0890	301.01	Isoquercitin	+	
6.08	302.01	300.9991	229.01	Ferulic acid	+	+
6.27	594.16	593.1479	285.05	Tribuloside		+
6.59	448.10	447.0962	286.03	Kaempferol hexoside	+	+
6.67	478.11	477.1068	315.04	Isorhamnetin-*O*-hexoside	+	
8.72	680.22	679.3714	517.31	Ferulic acid triglucoside	+	+

#### 3.1.1 Compound 3 (caffeic acid hexoside)

The TOF-MS/MS spectrum of compound **3** displayed a molecular ion peak at *m/z* 341.1070 [M-H]^-^ with deprotonated caffeic acid ion fragment at *m/z* 179.0542 [M-162]^-^ after losing 162 Da (hexose moiety) (Supplementary Figure S1) (Pacifico et al., 2019).

#### 3.1.2 Compound 8 (methoxy apigenin-*C*-pentoside)

The MS/MS fragmentation spectrum of compound **8** showed a molecular ion peak at *m/z* 431.1846. The peaks at *m/z* 299.9988 represented [M-H]^-^ and [M-132]^-^, respectively. A fragment ion peak was detected resulting from the loss of 132 Da corresponding to pentose moiety. It was attributed to methoxy apigenin, supporting the suggestion of the *O*-linkage of the sugar moiety (Supplementary Figure S3) (Zengin et al., 2021).

#### 3.1.3 Compound 13 (Quercetin pentose hexoside) and compound 16 (Isoquercitrin)

The MS/MS spectrum of compounds **13** and **16** displayed a deprotonated molecular ion at *m/z* 300.0291 corresponding to quercetin as the aglycone moiety in both compounds. It was created, by the loss of 132 Da (dehydrated pentose moiety) and 162 Da (dehydrated hexose moiety) from the main peak of **13** at *m/z* 595.1299 [M-H]^-^ (Supplementary Figure S4) (Unuofin and Lebelo, 2021) while in case of **16**, it resulted from the neutral loss of 162 Da (dehydrated hexose moiety) from the parent ion *m/z* 463.0890 [M-H]^-^ (Supplementary Figure S5) (Zengin et al., 2021).

#### 3.1.4 Compound 4 (Kaempferol hexoside) and compound 18 (Tribuloside)

The presence of kaempferol aglycone was suggested in both compounds since their MS/MS revealed a deprotonated molecular ion peak at *m/z* 285.0393 for kaempferol moiety which resulted from the loss of neutral 308 Da (dehydrated coumaroyl hexose moiety) from the main peak at *m/z* 593.1479 [M-H]^-^ (Unuofin and Lebelo, 2021) in compound **18** (Supplementary Figure S7), as well as, due to the loss of neutral 162 Da (dehydrated hexose moiety) from the parent peak at *m/z* 447.0962 [M-H]^-^ for compound (**4**) (Supplementary Figure S2) (Formato et al., 2021).

#### 3.1.5 Compound 17, ellagic acid

The MS/MS (Supplementary Figure S6) spectrum of compound **17** displays the molecular ion peak at *m/z* 300.9991 [M-H]^-^ with a daughter peak at *m/z* 229.0138 [M-H]^-^ consistent with the loss of CO_2_ and CO groups (77 Da) (Yan et al., 2014) (Supplementary Figure S6).

#### 3.1.6 Compound 22 (Isprhamnetin)

Compound **22** was identified based on its MS/MS data which displays a molecular ion peak at *m/z* 477.1068 [M-H]^-^ with a fragment peak at *m/z* 314.0459 [M-162]^-^ due to the loss of hexose moiety (162 Da) (Romussi et al., 1988; Jiang et al., 2017) (Supplementary Figure S8).

#### 3.1.7 Compound 25 (Ferulic acid triglucoside)

In compound **25**, the loss of 162 Da (hexose moiety) from the molecular ion peak 679.3714 [M-H]^-^ produced a fragment ion at *m/z* 517.3174 [M-162]^-^ (Kulshreshtha and Rastogi, 1971; Usui et al., 2017) (Supplementary Figure S9).

### 3.2 *In vivo* anti-diarrheal activity

#### 3.2.1 Acute toxicity study

The dose of 5 g/kg of both extracts, which is the maximum tested dose in the acute study, did not reveal any mortality or change in the behavior of the animals. Therefore, the selection of the AME at a dose of 250, 500, and 1,000 mg/kg was considered to be safe and this result was agreeing with previously reported LD_50_ for *Q. robur* (Moharram et al., 2015).

#### 3.2.2 Castor oil–induced diarrheal model

The obtained data showed that *Q. robur* and *Q. coccinea* AME significantly delay in a dose-dependent manner the onset of diarrhea (Table 3), in addition they decrease (*p* < 0.05) the total number of wet feces following castor oil administration comparing to the control and treated groups, especially in the case of *Q. robur* AME at 500 (1.83 ± 0.17) (*p* < 0.001), and 1,000 (1.50 ± 0.22) (*p* < 0.0001). In addition, *Q. robur* AME significantly decreased the average total feces number in comparison with the control at 250, 500, and 1,000 mg/kg b. wt (*p* < 0.0001). The highest defecation inhibition percentage was also detected with *Q. robur* AME at 1,000 mg/kg (57.14) which was non-significant from loperamide as the antidiarrheal drug (66.66).

**TABLE 3:** Effects of *Q. coccinea* and *Q.robur* AME on fecal characteristics in castor oil-induced diarrhea in mice.

Groups	Doses (mg/kg)	Onset of diarrhea	Number of normal feces	Number of wet feces	Number of watery feces	Total feces number	% of diarrheal inhibition
Control		63.00 ± 5.63	0.67 ± 0.21	3.50 ± 0.22	7.17 ± 0.31	11.33 ± 0.33	—
Loperamide	5	170.03 ± 1.28^a^	3.50 ± 0.22^a^	1.17 ± 0.31^a^	1.33 ± 0.21^a^	6.17 ± 0.31^a^	66.66
*Q. coccinea*	250	74.17 ± 2.95^b^	1.00 ± 0.00^b^	2.67 ± 0.21^b^	3.83 ± 0.40^a^ ^,^ ^b^	7.17 ± 0.48^a^	23.8
	500	89.83 ± 2.98^a^ ^,^ ^b^	1.67 ± 0.21^b^	2.50 ± 0.26^b^	3.00 ± 0.37^a^ ^,^ ^b^	7.00 ± 0.26^a^	28.57
	1,000	112.2 ± 2.86^a^ ^,^ ^b^	2.67 ± 0.21^a^	2.00 ± 0.22^a^	2.50 ± 0.22^a^	6.83 ± 0.60^a^	42.86
*Q.robur*	250	87.33 ± 0.79^a^ ^,^ ^b^	2.00 ± 0.37^a^ ^,^ ^b^	2.33 ± 0.21^b^	2.67 ± 0.21^a^ ^,^ ^b^	7.00 ± 0.52^a^	33.34
	500	111.7 ± 9.71^a^ ^,^ ^b^	2.50 ± 0.22^a^	1.83 ± 0.17^a^	1.67 ± 0.21^a^	5.50 ± 0.43^a^	47.3
	1,000	152.5 ± 0.93^a^	3.00 ± 0.37^a^	1.50 ± 0.22^a^	1.50 ± 0.22^a^	5.83 ± 0.48^a^	57.14

Data presented as: Mean ± SEM (*n* = 6);

^a^ significant from control

^b^ significant from loperamide.

#### 3.2.3 Castor oil-induced enter-pooling activity

After treatment with AME of *Q. robur* and *Q. coccinea,* it was found that they exerted in a dose dependent manner a significant suppression of castor oil-induced enter pooling (*p* < 0.001) compared with the untreated group (Table 4). Accordingly, after measuring the intestinal fluid volume, they were 0.48, 0.38, and 0.31 mL for 250, 500, and 1,000 mg/kg b. wt respectively with *Q. robur* AME and 0.57, 0.47, and 0.39 mL for 250, 500, and 1,000 mg/kg b. wt respectively with *Q. coccinea* AME. The results were compared with 0.78 and 0.28 mL for the untreated control and loperamide groups, respectively.

**TABLE 4:** Effects of *Q. coccinea* and *Q. robur* AME on castor oil-induced enter pooling.

Group	Dose (mg/kg)	Volume of intestinal fluid (mL)	% of inhibition
Control		0.78 ± 0.031	—
Loperamide	5	0.28 ± 0.012^a^ ^,^ ^c^ ^,^ ^d^	64.52
AME of *Q. coccinea*	250	0.57 ± 0.019^a^ ^,^ ^b^ ^,^ ^c^ ^,^ ^d^ ^,^ ^e^	27.09
	500	0.47 ± 0.013^a^ ^,^ ^b^ ^,^ ^d^ ^,^ ^e^	39.78
	1,000	0.39 ± 0.010^a^ ^,^ ^b^ ^,^ ^c^ ^,^ ^e^	50.10
AME of *Q. robur*	250	0.48 ± 0.014^a^ ^,^ ^b^ ^,^ ^d^ ^,^ ^e^	38.71
	500	0.38 ± 0.011^a^ ^,^ ^b^ ^,^ ^c^	51.19
	1,000	0.31 ± 0.009^a^ ^,^ ^c^	60

Data presented as: Mean ± SEM (*n* = 6).

^a^ Significant from control

^b^ Significant from loperamide

^c^ Significant from *Q. robur* 250 mg

^d^ Significant from *Q. robur* 500 mg

^e^ Significant from *Q. robur* 1,000 mg.

#### 3.2.4 Castor oil triggered charcoal meal transit test

The results showed that *Q. robur* and *Q. coccinea* AME suppressed gastrointestinal transit movement and affected peristaltic index (*p* < 0.0001) in a dose-dependent manner, which was confirmed by decreasing the mean of the distance moved by charcoal (*p* < 0.0001) compared with the control groups (Table 5). Furthermore, *Q. robur* showed a non-significant change from the standard group at 1,000 mg/kg.

**TABLE 5:** Effects of *Q. coccinea* and *Q. robur* on gastrointestinal motility in mice.

Group	Dose (mg/kg)	Length of small intestine (cm)	Distance traveled by charcoal (cm)	Peristaltic index (%)	% of inhibition
Control		52.83 ± 0.75	34.83 ± 0.60	66.01 ± 1.65	—
Loperamide	5	53.83 ± 0.70	12.33 ± 0.33^a^ ^,^ ^c^ ^,^ ^d^	22.95 ± 0.81^a^ ^,^ ^c^ ^,^ ^d^	65.23
AME of *Q. coccinea*	250	52.50 ± 1.06	28.00 ± 0.63^a^ ^,^ ^b^ ^,^ ^c^ ^,^ ^d^ ^,^ ^e^	53.48 ± 1.88^a^ ^,^ ^b^ ^,^ ^d^ ^,^ ^e^	18.98
	500	53.33 ± 0.56	25.17 ± 0.70^a^ ^,^ ^b^ ^,^ ^d^ ^,^ ^e^	47.18 ± 1.17^a^ ^,^ ^b^ ^,^ ^d^ ^,^ ^e^	28.53
	1,000	54.00 ± 0.58	22.83 ± 0.83^a^ ^,^ ^b^ ^,^ ^d^ ^,^ ^e^	42.28 ± 1.46^a^ ^,^ ^b^ ^,^ ^d^ ^,^ ^e^	35.95
AME of *Q.robur*	250	52.67 ± 1.26	25.00 ± 0.58^a^ ^,^ ^b^ ^,^ ^d^ ^,^ ^e^	47.71 ± 2.16^a^ ^,^ ^b^ ^,^ ^d^ ^,^ ^e^	27.72
	500	53.17 ± 0.60	19.67 ± 0.56^a^ ^,^ ^b^ ^,^ ^c^ ^,^ ^e^	37.04 ± 1.33^a^ ^,^ ^b^ ^,^ ^c^ ^,^ ^e^	43.89
	1,000	53.00 ± 0.89	14.00 ± 0.68^a^ ^,^ ^c^ ^,^ ^d^	26.41 ± 1.22^a^ ^,^ ^c^ ^,^ ^d^	59.99

Data presented as: Mean ± SEM (*n* = 6)

^a^ Significant from control

^b^ Significant from loperamide

^c^ Significant from *Q.robur* 250 mg

^d^ Significant from *Q. robur* 500 mg

^e^ Significant from *Q. robur* 1,000 mg.

## 4 Discussion

Hydro-methanol solution (80% aqueous methanol) can extract non-polar and polar phytochemicals (Zayede et al., 2020) so we used it to extract the polyphenolic constituents from both *Q. coccinea* and *Q. robur* leaves as well as to tentative identify the polyphenolic constituents by UHPLC/MS. The antidiarrheal activity of both AME was assessed *in vivo* in three diarrhea models using castor oil as a stimulant laxative. Ricinoleic acid, the pharmacologically active component present in castor oil is liberated by the effect of the lipase enzyme present in the small intestine’s upper part (Kulkarni and Pandit, 2005). Ricinoleic acid binds and activates the prostanoid EP3 receptor and motivates the endogenous prostaglandins synthesis from the intestinal arachidonic acid. Prostaglandins can advance gastrointestinal motility, exert a laxative effect, and modify the electrolytes and water movement in and out the lumen of the intestine (Tunaru et al., 2012; Adeniyi et al., 2017). Consequently, the use of castor oil to encourage diarrhea is similar to diarrhea pathophysiology, which rationalizes its use in the current research. Previous reports revealed that the anti-diarrheal effect of traditional medicinal plants is usually associated to their secondary metabolites as flavonoids, and tannins (Meite et al., 2009; Emudainohwo et al., 2015). Plants used to tackle diarrhea were found to exert their effect through different mechanisms including antiparasitic effect, suppression of gastric motility, and peristaltic index (Özbilgin et al., 2013). Our results revealed that *Q. robur* and *Q. coccinea* AME displayed a significant result on all measured parameters including the diarrhea onset, and the number of wet, watery, and total feces which indicated that they exert their antidiarrheal through an antisecretory mechanism. It is worth mentioning that the flavonoids, phenolic acids, and tannins were detected in the HPLC/MS of the AME which are well-known for their antidiarrheal effect *via* prostaglandin production inhibition (Hämäläinen et al., 2011). Furthermore, previous reports revealed that tannins have the ability to form complex with protein that help in the precipitation of the intestinal mucosa proteins making it more resistance to alteration by chemicals and consequently reduce intestinal secretion and peristaltic movements (Kumar and Upadhyaya, 2012). Another antidiarrheal mechanism for the drug is manifested by reducing the propulsive movement of the gastrointestinal smooth muscles. Consequently, enter pooling and motility tests were assessed to give more evidence for the antidiarrheal mechanism of the extract. In the castor oil-induced enter pooling method, *Q. robur* and *Q. coccinea* AME significantly decreased the intraluminal fluid accumulation compared with the control group. Meanwhile, castor oil improves prostaglandins production which motivates fluid secretion by avoiding the reabsorption of water and electrolytes (Pierce et al., 1971). The AME of both species inhibited the gastrointestinal hypersecretion and enter pooling *via* increasing reabsorption of water and electrolytes otherwise through the inhibition of the induced intestinal fluid accumulation which again could be attributed to their polyphenolic content. Additionally, the method of the charcoal meal was measured to explain another antidiarrheal mechanism by monitoring gastrointestinal content transposition because of gastrointestinal motility reduction (Qnais et al., 2005; Ezekwesili-Ofili et al., 2016). The current results suggested that the AME of both species significantly inhibited the intestinal transit by reducing the gastrointestinal motility of charcoal meal, which led to an increase in the intestinal content passage time therefore it was suggested that the AME act on all intestinal parts as well as its anti-motility property (Islam et al., 2013).

Our results revealed that the two Quercus species are rich in flavonoids which are supposed to inhabit intestinal motility, water, and electrolyte secretions by interfering with the auto-acid and prostaglandins activity (Tiwari et al., 2011). Our results revealed also the presence of quercetin and its glycosides which yield quercetin in the intestine and it was reported that the plants containing quercetin, can produce antidiarrheic effects mainly due to its antihistamine and anti-inflammatory activities (Carlo et al., 1994) also other reports revealed that quercetin exerts its antidiarrheic action through inhibition of gastrointestinal release of acetylcholine (Lutterodt, 1989; Perrucci et al., 2006). Moreover, our result revealed the presence of hydrolyzable tannins which are recognized for their antidiarrheal activity through denaturing intestinal mucosa proteins by forming protein tannate, which makes them stronger to chemical changes and so reduces secretion (Sardans et al., 2014; Sanda et al., 2020). In addition, tannins possess an anti-motility activity due to their anti-spasmodic effect, which inhibits secretion and intestinal motility *via* decreasing Ca^2+^ influx or improving calcium outflow (Bamisaye et al., 2013). Moreover, the presence of gallic acid or its derivatives (strictinin and tetra galloyl glucose) which produce gallic acid on hydrolysis in the extract may enhance the antidiarrheal activity since gallic acid has anti-secretory activity through inhibition of H ^+^ K ^+^ -ATPase activity (Chen et al., 2006), moreover it was reported that ellagic acid possesses antidiarrheal activity (Xiao et al., 2013). Therefore, it is speculated that the effectiveness of crude extract may be owing to the interaction between the different constituents in the extract leading to good activity and/or a decrease in possible toxicity of some distinct compounds. Otherwise, the individual action of different constituents found in the extract may collectively contribute to the efficacy of the extract. This finding justifies the ethnomedicinal use of the *Q. coccinea* and *Q. robur* extract in the treatment of diarrheal.

## 5 Conclusion

The current results revealed that the *Q. robur* and *Q. coccinea* leaves extracts showed antidiarrheal activity, which could be responsible for their inhibitory effect on gastrointestinal motility, and secretion. The secondary metabolites identified in the extracts mainly flavonoids and tannins could be owing to the Quercus species’ antidiarrheal effect through different mechanisms. Conversely, more research is required to establish the mechanism of action for antidiarrheal activity and to purify the active compounds responsible for the activity. Moreover, the research results gave evidence for the traditional uses of *Quercus* species and direct that they can be used for the treatment of diarrhea.

## Data Availability

The datasets presented in this study can be found in online repositories. The data presented in the study are deposited in the https://doi.org/10.6084/m9.figshare.21829956.v2 repository Supplementary Material.

## Abbreviations

Polyphenols, They are a large classis of the plant secondary metabolites which possess number of hydroxyl groups on aromatic ring and they exerts a wide variety of biological activity; UHPLC-MS analysis, High-performance liquid chromatography coupled with mass spectroscopy technique is used to separate, identify and quantify different compounds present in a mixture. The median lethal dose: (LD_50_), The dose of compounds or extract lethal for 50% of the experiential animals; *In vivo* study, It means that the research work is done with or within an entire, living organism; Gavage, Introduction of the drug into the stomach using oral tube; Peristaltic index, Percentage of the distance travelled by the charcoal meal relative to the total length of the small intestine; Diarrhea, Is the frequent passage of a watery movable stool; Anti-diarrheal drug, Are the drugs which control diarrhea and decrease the passage of stool through the intestine; Hydrolyzable tannins, Are a class of compounds containing glucose or polyol central core esterified with gallic acid (gallotannins) or with hexahydroxydiphenic acid (ellagitannins); Proanthocyanidins, They are oligomeric and polymeric products of the flavonoid biosynthetic pathway and their building units are catechin and epicatechin; Galls, Is a large, round, indefinitely apple-like, it is commonly found on many Quercus species. Galls are result due to chemicals injected by the larva of certain kinds of gall wasp in the family Cynipidae.

## References

[B1] Abdela J. (2019). Evaluation of *in vivo* antidiarrheal activities of hydroalcoholic leaf extract of *Dodonaea viscosa* L.(Sapindaceae) in Swiss albino mice. J. Evid. Based Integr. Med. 24, 2515690X19891952. 10.1177/2515690X19891952 PMC6918490 31840545

[B2] Adeniyi O. S. Omale J. Omeje S. C. Edino V. O. (2017). Antidiarrheal activity of hexane extract of *Citrus limon* peel in an experimental animal model. J. Integr. Med. 15, 158–164. 10.1016/S2095-4964(17)60327-3 28285621

[B3] Agbor G. A. Léopold T. Jeanne N. Y. (2004). The antidiarrhoeal activity of *Alchornea cordifolia* leaf extract. Phytother. Res. 18, 873–876. 10.1002/ptr.1446 15597302

[B4] Amr A. S. Ahmad M. N. Zahra J. A. Abdullah M. A. (2021). HPLC/MS-MS identification of oak *Quercus aegilops* root tannins. J. Chem. 2021, 1–10. 10.1155/2021/8882050

[B5] Arámbula-Salazar J. Almaraz-Abarca N. Corral-Rivas J. J. Delgado A. Moreno R. Montiel-Antuna E. (2015). Variability in foliar phenolic composition of several *quercus* species in northern Mexico. Pak. J. Sci. Ind. Res. B Biol. Sci. 58, 79–89. 10.52763/PJSIR.PHYS.SCI.58.2.2015.79.89

[B6] Aung T. Bibat M. a. D. Zhao C.-C. Eun J.-B. (2020). Bioactive compounds and antioxidant activities of *Quercus salicina* Blume extract. Food Sci. Biotechnol. 29, 449–458. 10.1007/s10068-020-00755-1 32296555 PMC7142199

[B7] Bamisaye F. Odutuga A. A. Minari J. Dairo J. O. Fagbohunka B. Oluba O. (2013). Phytochemical constituents and antidiarrhoeal effects of the aqueous extract of *Terminalia superba* leaves on Wistar rats. Afr. J. Pharm. Pharmacol. 7, 848–851. 10.5897/AJPP12.332

[B8] Behera K. K. Mandal P. Mahapatra D. J. E. L. (2006). Green leaves for diarrhoeal diseases used by the tribals of kenojhar and mayurbhanj district of Orissa, India. Ethnobot. Leafl. 10, 305–328.

[B9] Belemtougri R. Constantin B. Cognard C. Raymond G. Sawadogo L. (2006). Effects of two medicinal plants *Psidium guajava* L. (Myrtaceae) and *Diospyros mespiliformis* L. (Ebenaceae) leaf extracts on rat skeletal muscle cells in primary culture. J. Zhejiang Univ. Sci. B 7, 56–63. 10.1631/jzus.2006.B0056 16365927 PMC1361761

[B10] Brijesh S. Daswani P. Tetali P. Antia N. Birdi T. (2009). Studies on the antidiarrhoeal activity of *Aegle marmelos* unripe fruit: Validating its traditional usage. BMC Complement. Altern. Med. 9, 47. 10.1186/1472-6882-9-47 19930633 PMC2788518

[B11] Burlacu E. Nisca A. Tanase C. (2020). A comprehensive review of phytochemistry and biological activities of *quercus* species. Forests 11, 904. 10.3390/f11090904

[B12] Burns R. M. Honkala B. H. (1990). Silvics of North America. Washington, DC: United States Department of Agriculture.

[B13] Bursal E. Boğa R. (2018). Polyphenols analysed by UHPLC-ESI-MS/MS and antioxidant activities of molasses, acorn and leaves of oak (*Quercus robur* subsp. pedunculiflora). Prog. Nutr. 20, 167–175. 10.23751/pn.v20i1-S.5311

[B14] Carlo G. D. Mascolo N. Izzo A. A. Capasso F. Autore G. (1994). Effects of quercetin on the gastrointestinal tract in rats and mice. Phytother. Res. 8, 42–45. 10.1002/ptr.2650080110

[B15] Chen J.-C. Ho T.-Y. Chang Y.-S. Wu S.-L. Hsiang C.-Y. (2006). Anti-diarrheal effect of Galla Chinensis on the *Escherichia coli* heat-labile enterotoxin and ganglioside interaction. J. Ethnopharmacol 103, 385–391. 10.1016/j.jep.2005.08.036 16213682

[B16] Chitme H. R. Chandra M Fau - Kaushik S. Kaushik S. (2004). Studies on anti-diarrhoeal activity of *Calotropis gigantea* R.Br. in experimental animals. J. Pharm. Pharm. Sci. 7, 70–75. 15144737

[B17] Dar Ms Fau - Ikram M. Ikram M. (1979). Studies on *Quercus infectoria*; isolation of syringic acid and determination of its central depressive activity. Planta Med. 35, 156–161. 10.1055/s-0028-1097197 419182

[B18] Deveoglu O. Torgan E. Karadag R. (2012). Identification by rp-hplc-dad of natural dyestuffs from lake pigments prepared with a mixture of weld and dyer's oak dye plants. J. Liq. Chromatogr. Relat. Technol. 35, 331–342. 10.1080/10826076.2011.601487

[B19] Dróżdż P. Pyrzynska K. (2018). Assessment of polyphenol content and antioxidant activity of oak bark extracts. Eur. J. wood wood Prod. 76, 793–795. 10.1007/s00107-017-1280-x

[B20] Eaton E. Caudullo G. Oliveira S. De Rigo D. (2016). “Quercus robur and quercus petraea in Europe: Distribution, habitat, usage and threats,” in European atlas of forest tree species. Editors San-Miguel-Ayanz J. de Rigo D. Caudullo G. Houston Durrant T. Mauri A. (Luxembourg: Publication Office of the European Union), 160–163.

[B21] Elsayed H. E. Ebrahim H. Y. Mady M. S. Khattab M. A. El-Sayed E. K. Moharram F. A. (2022). Ethnopharmacological impact of *Melaleuca rugulosa* (Link) Craven leaves extract on liver inflammation. J. Ethnopharmacol. 292, 115215. 10.1016/j.jep.2022.115215 35337921

[B22] Emudainohwo J. O. T. Earnest E. Moke E. (2015). Anti-diarrheal activity of the aqueous leaf extract of *ageratum conyzoides* in wistar rats. J. Appl. Sci. Environ. Manag. 19, 169. 10.4314/jasem.v19i2.1

[B23] Ezekwesili-Ofili J. Nkemdilim U. U. Okeke C. (2016). Mechanism of antidiarrhoeal effect of ethanolic extract of *Psidium guajava* leaves. Biokemistri 22, 85–90.

[B24] Farag S. F. El-Emary N. A. Niwa M. (1998). Gallotannins from *Quercus robur* cultivated in Egypt. J Bull. Pharm. Sci. Assiut 21, 1–6. 10.21608/bfsa.1998.67765

[B25] Formato M. Piccolella S. Zidorn C. Pacifico S. (2021). UHPLC-HRMS analysis of *Fagus sylvatica* (Fagaceae) leaves: A renewable source of antioxidant polyphenols. Antioxidants 10, 1140. 10.3390/antiox10071140 34356373 PMC8301150

[B26] Formato M. Vastolo A. Piccolella S. Calabrò S. Cutrignelli M. I. Zidorn C. (2022). Antioxidants in animal nutrition: UHPLC-ESI-QqTOF analysis and effects on *in vitro* rumen fermentation of oak leaf extracts. Antioxidants 11, 2366. 10.3390/antiox11122366 36552573 PMC9774136

[B27] Gao Z. Wei Z. Zhang J. Su Y. (2021). Chemical constituents of the seeds of *Quercus wutaishanica* . Chem. Nat. Compd. 57, 650–653. 10.1007/s10600-021-03442-7

[B28] Grieve M. (1984). A modern herbal: The medicinal, culinary, cosmetic and economic properties, cultivation and folk lore of herbs, grasses, fungi, shrubs and trees. London.: Penguin Books.

[B29] Guerrant R. L. Van Gilder T. Steiner T. S. Thielman N. M. Slutsker L. Tauxe R. V. (2001). Practice Guidelines for the management of infectious diarrhea. Clin. Infect. Dis. 32, 331–351. 10.1086/318514 11170940

[B30] Hämäläinen M. Nieminen R. Asmawi M. Vuorela P. Vapaatalo H. Moilanen E. (2011). Effects of flavonoids on prostaglandin E-2 production and on COX-2 and mPGES-1 expressions in activated macrophages. Plant Med. 77, 1504–1511. 10.1055/s-0030-1270762 21341175

[B31] Herve Du Penhoat C. L. M. Michon V. M. F. Ohassan A. Peng S. Scalbert A. Gage D. (1991). Roburin A, A dimeric ellagitannin from heartwood of *Quercus robur* . Phytochem 30, 329–332. 10.1016/0031-9422(91)84148-L

[B32] Holopainen J. K. Virjamo V. Ghimire R. P. Blande J. D. Julkunen-Tiitto R. Kivimäenpää M. (2018). Climate change effects on secondary compounds of forest trees in the northern hemisphere. Front. Plant Sci. 9, 1445. 10.3389/fpls.2018.01445 30333846 PMC6176061

[B33] Islam M. M. Rashna S. P. Kazi S. E. Jesmin C. Fahima A. Nahida P. (2013). Antidiarrheal activity of *Dillenia indica* bark extract. Int. J. Pharm. Sci. Res. 4, 682–688. 10.13040/IJPSR.0975-8232.4(2).682-88

[B34] Jaradat N. A. Ayesh O. I. Anderson C. (2016). Ethnopharmacological survey about medicinal plants utilized by herbalists and traditional practitioner healers for treatments of diarrhea in the West Bank/Palestine. J. Ethnopharmacol. 182, 57–66. 10.1016/j.jep.2016.02.013 26883246

[B35] Jiang Z. Wang J. Chen X. Wang X. Wang T. Zhu Z. (2017). Simultaneous determination of kaempferide, kaempferol and isorhamnetin in rat plasma by ultra-high performance liquid chromatography-tandem mass spectrometry and its application to a pharmacokinetic study. J. Braz. Chem. Soc. 29. 10.21577/0103-5053.20170166

[B36] Kulkarni S. R. Pandit A. (2005). Enzymatic hydrolysis of castor oil: An approach for rate enhancement and enzyme economy. Indian J. Biotechnol. 4, 241–245.

[B37] Kulshreshtha D. K. Rastogi R. P. (1971). Chemical constituents of *Quercus lanceaefolia* . Phytochem 10, 2831–2832. 10.1016/S0031-9422(00)97298-4

[B38] Kumar P. Upadhyaya K. (2012). Tannins are astringent. J. Pharmacogn. Phytochem. 1, 45–50.

[B39] Lozoya X. Reyes-Morales H. Chávez-Soto M. A. Martínez-García M. Soto-González Y. Doubova S. V. (2002). Intestinal anti-spasmodic effect of a phytodrug of *Psidium guajava* folia in the treatment of acute diarrheic disease. J. Ethnopharmacol. 83, 19–24. 10.1016/S0378-8741(02)00185-X 12413703

[B40] Lutterodt G. D. (1989). Inhibition of gastrointestinal release of acetylchoune byquercetin as a possible mode of action of *Psidium guajava* leaf extracts in the treatment of acute diarrhoeal disease. J. Ethnopharmacol 25, 235–247. 10.1016/0378-8741(89)90030-5 2747259

[B41] Mady M. S. Elsayed H. E. El-Sayed E. K. Hussein A. A. Ebrahim H. Y. Moharram F. A. (2022). Polyphenolic profile and ethno pharmacological activities of *Callistemon subulatus* (Cheel) Craven leaves cultivated in Egypt. J. Ethnopharmacol. 284, 114698. 10.1016/j.jep.2021.114698 34600075

[B42] Mahgoub S. Hashad N. Ali S. Ibrahim R. Said A. M. Moharram F. A. (2021). Polyphenolic profile of *Callistemon viminalis* aerial parts: Antioxidant, anticancer and in silico 5-LOX inhibitory evaluations. Molecules 26, 2481. 10.3390/molecules26092481 33923148 PMC8123052

[B43] Meite S. Jean David N. G. Bahi C. Yapi H. F. Djaman J. Guede-Guina F. (2009). Antidiarrheal activity of the ethyl acetate extract of *Morinda morindoides* in rats. Trop. J. Pharm. Res. 8, 201–207. 10.4314/tjpr.v8i3.44533

[B44] Mekonnen B. Asrie A. B. Wubneh Z. B. (2018). Antidiarrheal activity of 80% methanolic leaf extract of *Justicia schimperiana* . Evid.-based Complement. Altern. Med. 2018, 3037120. 10.1155/2018/3037120 PMC5818970 29541140

[B45] Meng Z. Zhou Y. Lu J. Sugahara K. Xu S. Kodama H. (2001). Effect of five flavonoid compounds isolated from *Quercus dentata* Thunb on superoxide generation in human neutrophils and phosphorylation of neutrophil proteins. Clin. Chim. Acta 306, 97–102. 10.1016/S0009-8981(01)00403-X 11282099

[B46] Moharram F. Marzouk M. S. El Dib R. Elshenawy S. Abdel-Rahman R. Ibrahim R. (2015). Hepatoprotective, Gastroprotective, Antioxidant activity and phenolic constituents of *Quercus robur* leaves. J. Pharm. Sci. Res. 7, 1055–1065.

[B47] Nishimura H. Nonaka G.-I. Nishioka I. (1986). Scyllo-quercitol gallates and hexahydroxydiphenoates from *quercus stenophylla* . Phytochem 25, 2599–2604. 10.1016/S0031-9422(00)84517-3

[B48] Nishimura H. Nonaka G.-I. Nishioka I. (1984). Seven quinic acid gallates from *Quercus stenophylla* . Phytochem 23, 2621–2623. 10.1016/S0031-9422(00)84112-6

[B49] Oben J. Assi S. E. Agbor G. Musoro D. F. (2006). Effect of *Eremomastax speciosa* on experimental diarrhoea. Afr. J. Tradit. Complement. Altern. Med. 3, 95–100.

[B50] Ou L. M. Seken A. Li K-A. Ji Z-H. Tian S-G. (2015). Simultaneous determination of gallic acid, methyl gallate and ellagic acid in gall of *Quercus infectoria* Olivier by HPLC. Int. J. Pharm. Res. 42, 637–641. 10.13220/j.cnki.jipr.2015.05.017

[B51] Özbilgin A. Durmuskahya C. Kilimcioğlu A. Kayalar H. Kurt O. Ermiş V. (2013). *In vitro* efficacy of quercus infectoria oliv. And *Achillea millefolium* L. Extracts against blastocystis spp. isolates. Kafkas Univ. Vet. Fak. Derg. 19, 511–516. 10.9775/kvfd.2012.8196

[B52] Pacifico S. Piccolella S. Nocera P. Tranquillo E. Dal Poggetto F. Catauro M. (2019). New insights into phenol and polyphenol composition of *Stevia rebaudiana* leaves. J. Pharm. Biomed. Anal. 163, 45–57. 10.1016/j.jpba.2018.09.046 30286435

[B53] Park K. (2021). Park’s textbook of preventive and social medicine. Jabalpur: India M/S Banarsidas Bharat Publishers.

[B54] Perrucci S. Fichi G. Buggiani C. Rossi G. Flamini G. (2006). Efficacy of mangiferin against Cryptosporidium parvum in a neonatal mouse model. Parasitol. Res. 99, 184–188. 10.1007/s00436-006-0165-4 16547730

[B55] Pierce N. F. Carpenter C. C. J. Elliott H. L. Greenough W. B. (1971). Effects of prostaglandins, theophylline, and cholera exotoxin upon transmucosal water and electrolyte movement in the *canine jejunum* . Gastroenterology 60, 22–32. 10.1016/S0016-5085(71)80003-3 5544090

[B56] Qnais E. Y. Abdulla F. Ghalyun Y. Y. (2005). Antidiarrheal effects of *Juniperus phoenicia* L. Leaves extract in rats. Pak. J. Biol. Sci. 8, 867–871. 10.3923/pjbs.2005.867.871

[B57] Rahman N. S. Md Salleh L. Majid F. A. Harisun Y. (2015). Quantification of gallic acid and tannic acid from *Quercus infectoria* (Manjakani) and their effects on antioxidant and antibacterial activities. Pertanika J. Sci. Technol. 23, 351–362.

[B58] Romussi G. Bignardi G. Pizza C. (1988). Constituents of cupuliferae, XII. Minor acylated flavonoids from *Quercus cerris* L. Eur. J. Org. Chem. 1988, 989–991. 10.1002/JLAC.198819881010

[B59] Sakar K. Sohretoglu D. Özalp M. Ekizoglu M. Piacente S. Pizza C. (2005). Polyphenolic compounds and antimicrobial activity of *Quercus aucheri* leaves. Turk. J. Chem. 29, 555–559.

[B60] Salem M. Elansary H. El Kelish A. Zeidler A. Ali H. El-Hefny M. (2016). Vitro bioactivity and antimicrobial activity of *Picea abies* and *Larix decidua* wood and bark extracts. BioResources 11, 9421–9437. 10.15376/biores.11.4.9421-9437

[B61] Sanda K. Sandabe U. Bulama I. Babakura M. Madziga H. Mbursa C. (2020). Phytochemistry and effects of ethanol leaf extract of *Meytenus senengalensis* (lam) on castor-oil induced diarrhoea in albino rats. Sahel J. Veterinary Sci. 17, 1–6. 10.54058/saheljvs.v17i1.89

[B62] Sardans J. Gargallo-Garriga A. Pérez-Trujillo M. Parella T. J. Seco R. Filella I. (2014). Metabolic responses of *Quercus ilex* seedlings to wounding analysed with nuclear magnetic resonance profiling. Plant Biol. 16, 395–403. 10.1111/plb.12032 23590498

[B63] Saunier A. Greff S. Blande J. D. Lecareux C. Baldy V. Fernandez C. (2022). Amplified drought and seasonal cycle modulate *Quercus pubescens* leaf metabolome. Metabolites 12, 307. 10.3390/metabo12040307 35448494 PMC9026387

[B64] Scalbert A. Haslam E. (1987). Polyphenols and chemical defence of the leaves of *Quercus robur* . Phytochem 26, 3191–3195. 10.1016/S0031-9422(00)82468-1

[B65] Scalbert A. Monties B. Favre J.-M. (1988). Polyphenols of *Quercus robur*: Adult tree and *in vitro* grown calli and shoots. Phytochem 27, 3483–3488. 10.1016/0031-9422(88)80753-2

[B66] Sinan K. I. Bene K. Zengin G. Diuzheva A. Jekő J. Cziáky Z. (2021). A comparative study of the HPLC-MS profiles and biological efficiency of different solvent leaf extracts of two african plants: *Bersama abyssinica* and *Scoparia dulcis* . Int. J. Environ. Res. Public Health 31, 285–297. 10.1080/09603123.2019.1652885 31411055

[B67] Sinan K. I. Chiavaroli A. Orlando G. Bene K. Zengin G. Cziáky Z. (2020). Biopotential of *Bersama abyssinica* fresen stem bark extracts: UHPLC profiles, antioxidant, enzyme inhibitory, and antiproliferative propensities. Antioxidants 9, 163. LID - 10.3390/antiox9020163 [doi] LID - 163. 10.3390/antiox9020163 32079363 PMC7094211

[B68] Şöhretoğlu D. Renda G. (2020). The polyphenolic profile of oak (*quercus*) species: A phytochemical and pharmacological overview. Phytochem. Rev. 19, 1379–1426. 10.1007/s11101-020-09707-3

[B69] Stefi A. L. Nikou T. Papadopoulou S. Kalaboka Μ. Vassilacopoulou D. Halabalaki M. (2022). The response of the laboratory cultivated *Quercus coccifera* plants to an artificial water stress. Plant Stress 4, 100077. 10.1016/j.stress.2022.100077

[B70] Taib M. Rezzak Y. Bouyazza L. Lyoussi B. (2020). Medicinal uses, phytochemistry, and pharmacological activities of *quercus* species. Evid.-based Complement. Altern. Med. 2020, 1920683. 10.1155/2020/1920683 PMC7415107 32802116

[B71] Thapar N. Sanderson I. R. (2004). Diarrhoea in children: An interface between developing and developed countries. Lancet 363, 641–653. 10.1016/S0140-6736(04)15599-2 14987892

[B72] Tiwari P. Kumar B. Kaur M. Kaur G. Kaur H. (2011). Phytochemical screening and extraction: A review. Int. Pharm. Sci. 1, 98–106.

[B73] Tunaru S. Althoff T. F. Nüsing R. M. Diener M. Offermanns S. (2012). Castor oil induces laxation and uterus contraction via ricinoleic acid activating prostaglandin EP3 receptors. PNAS 109, 9179–9184. 10.1073/pnas.1201627109 22615395 PMC3384204

[B74] Unuofin J. O. Lebelo S. L. (2021). UHPLC-QToF-MS characterization of bioactive metabolites from *Quercus robur* L. grown in South Africa for antioxidant and antidiabetic properties. Arab. J. Chem. 14, 102970. 10.1016/j.arabjc.2020.102970

[B75] Usui A. Matsuo Y. Tanaka T. Ohshima K. Fukuda S. Mine T. (2017). Ferulic acid esters of oligo-glucose from *Allium macrostemon* . Nat. Prod. Commun. 12, 89–91. 10.1177/1934578x1701200125 30549834

[B76] Venkatesan N. Thiyagarajan V. Narayanan S. Arul A. S R. Kumar S. (2005). Antidiarrheal potential of *Asparagus racemous* wild root extracts in laboratoire animals. J. Pharm. Pharm. Sci. 8, 39–46. 15946596

[B77] Vivas N. Laguerre M. Glories Y. Bourgeois G. Vitry C. (1995). Structure simulation of two ellagitannins from *Quercus robur* L. Phytochem 39, 1193–1199. 10.1016/0031-9422(95)00148-Z

[B78] Vong A. T. Chong H. a.-O. Lim V. a.-O. (2018). Preliminary study of the potential extracts from selected plants to improve surface cleaning. Plants (Basel) 7, 17. 10.3390/plants7010017 29509658 PMC5874606

[B79] Vovk I. Simonovska B. Andrenšek S. Vuorela H. Vuorela P. (2003). Rotation planar extraction and rotation planar chromatography of oak (*Quercus robur* L.) bark. J. Chromatogr. A 991, 267–274. 10.1016/S0021-9673(03)00271-1 12741604

[B80] Wingate D. Phillips S. F. Lewis S. J. Malagelada J.-R. Speelman P. Steffen R. (2001). Guidelines for adults on self-medication for the treatment of acute diarrhoea. Aliment. Pharmacol. Ther. 15, 773–782. 10.1046/j.1365-2036.2001.00993.x 11380315

[B81] Xiao H.-T. Tsang S.-W. Qin H.-Y. Choi F. F. K. Yang Z.-J. Han Q.-B. (2013). A bioactivity-guided study on the anti-diarrheal activity of Polygonum chinense Linn. J. Ethnopharmacol. 149, 499–505. 10.1016/j.jep.2013.07.007 23895917

[B82] Xu J. Wang X. Yue J. Sun Y. Zhang X. Zhao Y. (2018). Polyphenols from acorn leaves (*Quercus liaotungensis*) protect pancreatic beta cells and their inhibitory activity against α-glucosidase and protein tyrosine phosphatase 1B. Molecules 23, 2167. 10.3390/molecules23092167 30154343 PMC6225166

[B83] Yadav A. K. Tangpu V. (2007). Antidiarrheal activity of *lithocarpus dealbata*. And *urena lobata*. Extracts: Therapeutic implications. Pharm. Biol. 45, 223–229. 10.1080/13880200701213153

[B84] Yan L. Yin P. Ma C. Liu Y. (2014). Method development and validation for pharmacokinetic and tissue distributions of ellagic acid using ultrahigh performance liquid chromatography-tandem mass spectrometry (UPLC-MS/MS). Molecules 19, 18923–18935. 10.3390/molecules191118923 25412040 PMC6271459

[B85] Yang X. Yin Y. Feng L. Tang H. Wang F. (2019). The first complete chloroplast genome of *Quercus coccinea* (Scarlet Oak) and its phylogenetic position within Fagaceae. Mitochondrial DNA B Resour. 4, 3634–3635. 10.1080/23802359.2019.1677189 33366118 PMC7707449

[B86] Zayede D. Mulaw T. Kahaliw W. (2020). Antidiarrheal activity of hydromethanolic root extract and solvent fractions of *Clutia abyssinica* jaub. & spach. (Euphorbiaceae) in mice. Evid.-based Complement. Altern. Med. 2020, 5416749. 10.1155/2020/5416749 PMC7201494 32419812

[B87] Zengin G. Mahomoodally M. F. Sinan K. I. Ak G. Etienne O. K. Sharmeen J. B. (2021). Chemical composition and biological properties of two *jatropha* species: Different parts and different extraction methods. Antioxidants 10, 792. 10.3390/antiox10050792 34067702 PMC8156752

[B88] Zhao S.-S. Ma D.-X. Zhu Y. Zhao J.-H. Zhang Y. Chen J.-Q. (2018). Antidiarrheal effect of bioactivity-guided fractions and bioactive components of pomegranate (*Punica granatum* L.) peels. Neurogastroenterol. Motil. 30, e13364. 10.1111/nmo.13364 29717519

