# Supplementary material for: UHPLC-MS profiles and antidiarrheal activity of Quercus coccinea münchh. and Quercus robur L. employing in vivo technique
Source: Front Pharmacol. 2023 Feb 17;14:1120146. doi: 10.3389/fphar.2023.1120146 (PMC9982048; doi:10.3389/fphar.2023.1120146)
Supplement: Supplementary file 1 [file DataSheet1.pdf]

# UHPLC-MS profiles and antidiarrheal activity of *Quercus coccinea* Münchh. and *Quercus robur* L. employing *in vivo* technique

**Mohamed S. Mady [1,#], Reham R. Ibrahim [1,#], Elsayed K. El-Sayed [2], Mohamed El-Shazly[3], Lo-Yun Chen[4], Kuei-Hung Lai[4,5,6,*], Fatheya S. El Shaarawy [1,†,], Fatma A. Moharram [1,†]**

[1] Faculty of Pharmacy, Helwan University, Pharmacognosy Department, Cairo, 11795, Egypt.
[2] Faculty of Pharmacy, Helwan University, Pharmacology and Toxicology Department, Cairo, 11795, Egypt.
[3]Faculty of Pharmacy, Ain-Shams University, Pharmacognosy Department, Cairo 11566, Egypt
[4] College of Pharmacy, Taipei Medical University, Graduate Institute of Pharmacognosy, Taipei 11031, Taiwan.
[5] College of Pharmacy, Taipei Medical University, PhD Program in Clinical Drug Development of Herbal Medicine Taipei 11031, Taiwan.
[6] Taipei Medical University Hospital, Traditional Herbal Medicine Research Center, Taipei 11031, 29 Taiwan

# These authors share the first authorship
† These authors share the last authorship

*** Corresponding authors**

**Mohamed S. Mady**

*E-mail:* mohamedsaid_1985@pharm.helwan.edu.eg

**Kuei-Hung Lai**

*E-mail:* kueihunglai@tmu.edu.tw
**Keywords:** Antidiarrheal, polyphenolic compounds, *Quercus coccinea*, *Quercus rubur*, UHPLC-MS

**The data presented in the study are deposited in the https://doi.org/10.6084/m9.figshare.21829956.v2 repository.**

## ABSTRACT

**Introduction***:* *Quercus* L. genus (Oak) belongs to the family Fagaceae and their galls are used commercially in leather tanning, dyeing, and ink preparation. Several *Quercus* species were traditionally used to manage wound healing, acute diarrhea, hemorrhoid, and inflammatory diseases. The present study aims to investigate the phenolic content of the 80% aqueous methanol extract (AME) of *Q. coccinea* and *Q. robur* leaves as well as to assess their anti-diarrheal activity. **Methods**: Polyphenolic content of *Q. coccinea* and *Q. robur* AME were investigated using UHPLC/MS. The antidiarrheal potential of the obtained extracts was evaluated by conducting a castor oil-induced diarrhea *in-vivo* model, **Result and Discussion**: Twenty-five and twenty-six polyphenolic compounds were tentatively identified in *Q. coccinea* and *Q. robur* AME, respectively. The identified compounds are related to quercetin, kaempferol, isorhamnetin, and apigenin glycosides and their aglycones. In addition, hydrolyzable tannins, phenolic acid, phenyl propanoides derivatives, and cucurbitacin F were also identified in both species AME of *Q. coccinea* (250, 500, and 1000 mg/kg) exhibited a significant prolongation in the onset of diarrhea by 17.7 %, 42.6%, and 79.7% respectively while AME of *Q robur* at the same doses significantly prolonged the onset of diarrhea by 38.6%, 77.3%, and 2.4 folds respectively as compared to the control. Moreover, the percentage of diarrheal inhibition of *Q. coccinea* was 23.8%, 28.57%, and 42,86% respectively, and for *Q robur* 33.34%, 47.3%, and 57.14% respectively as compared to the control group. Both extracts significantly decreased the volume of intestinal fluid by 27%, 39.78%, and 50.1% for *Q coccinea* respectively; and by 38.71%, 51.19%, and 60% for *Q robur* respectively as compared to the control group. In addition, AME of *Q. coccinea* exhibited a peristaltic index of 53.48, 47.18, and 42.28 with significant inhibition of gastrointestinal transit by 18.98%, 28.53%, and 35.95 % respectively; while AME of *Q robur* exhibited a peristaltic index of 47.71, 37, and 26.41 with significant inhibition of gastrointestinal transit by 27.72%, 43.89%, and 59.99% respectively as compared with the control group. Notably, *Q. robur* showed a better antidiarrheal effect in comparison with *Q. coccinea* and, the highest effect was observed for *Q. robur* at 1000 mg/kg as it was nonsignificant from the loperamide standard group in all measured parameters.

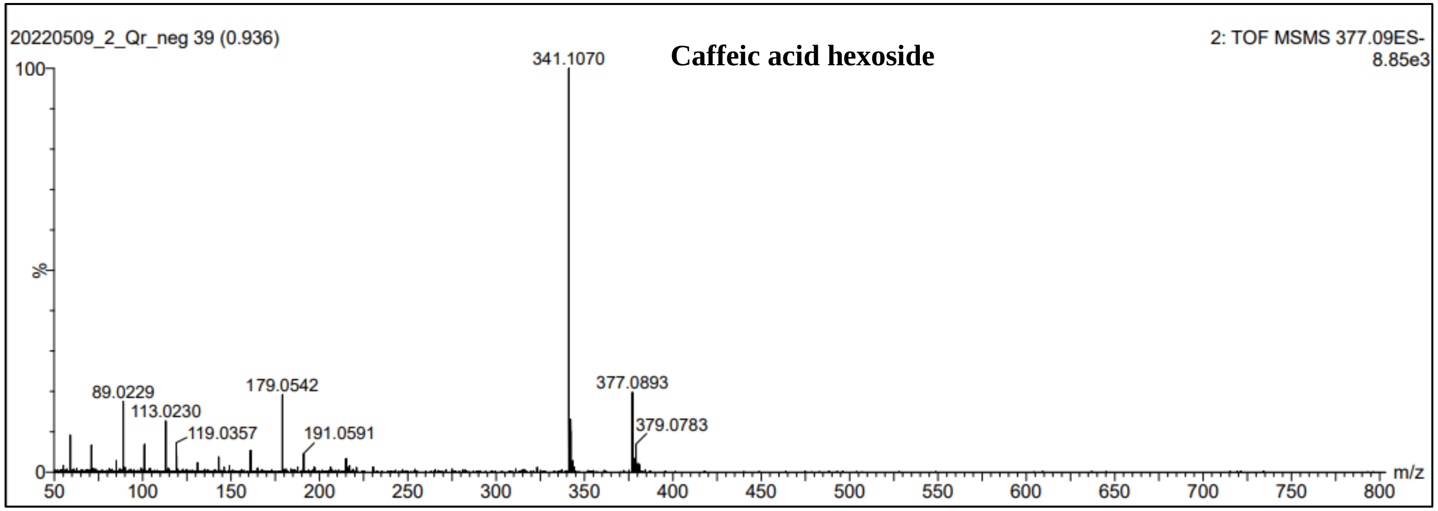


**Figure S1. TOF-MS/MS spectra of deprotonated compounds 3**

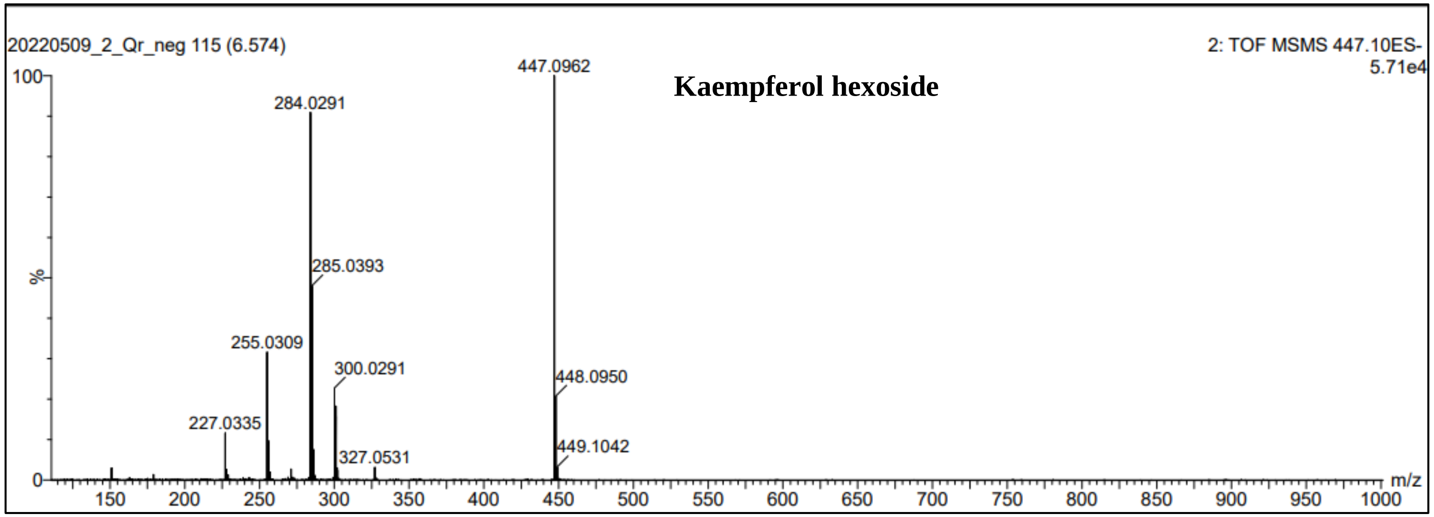

**Figure S2. TOF-MS/MS spectra of deprotonated compounds 4**

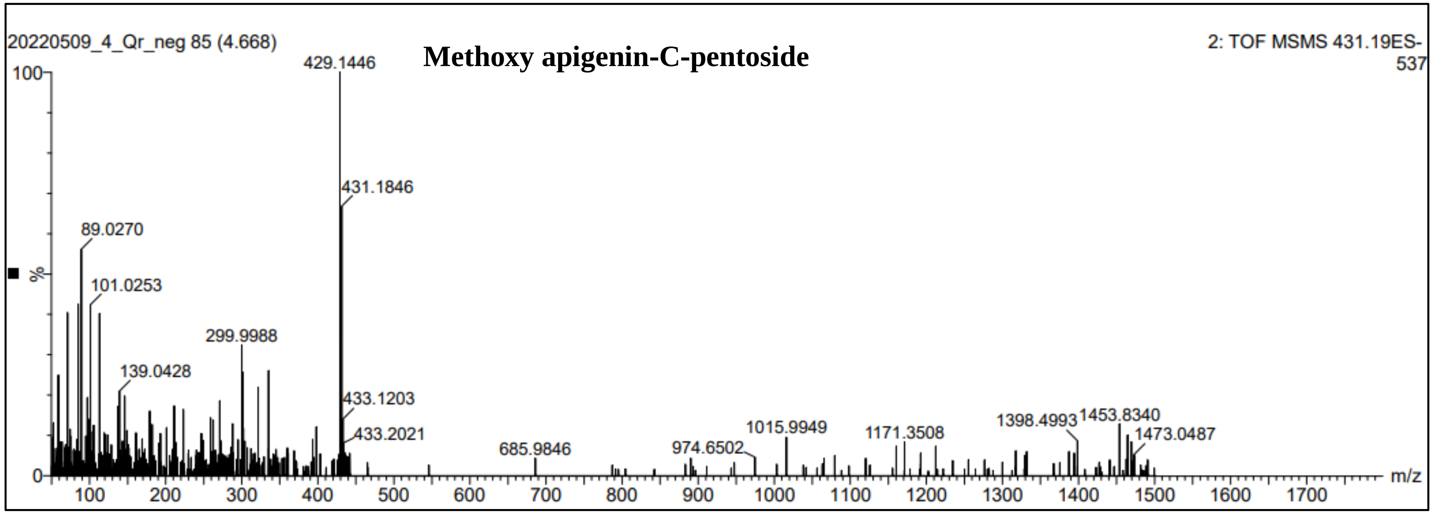


**Figure S3. TOF-MS/MS spectra of deprotonated compounds 8**

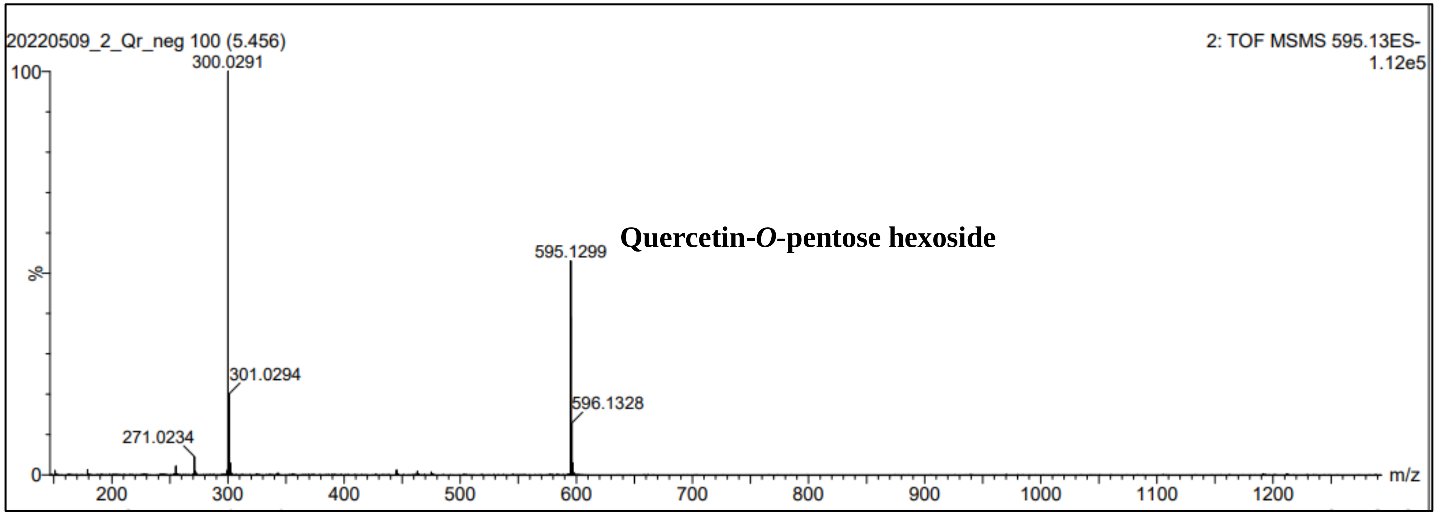

**Figure S4. TOF-MS/MS spectra of deprotonated compounds 13**

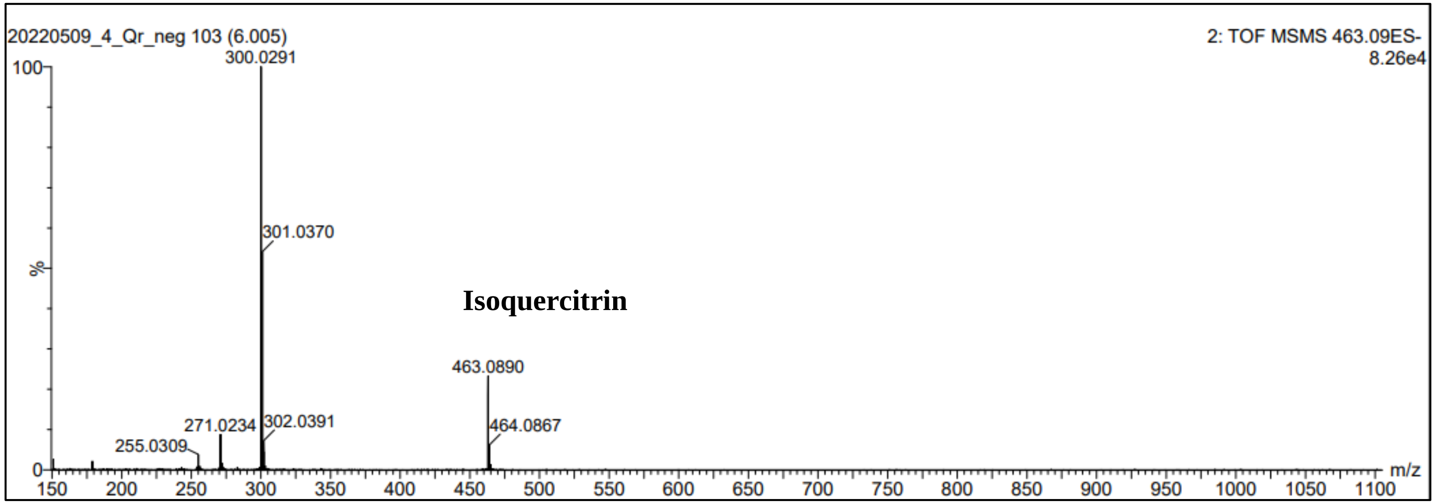


**Figure S5. TOF-MS/MS spectra of deprotonated compounds 16**

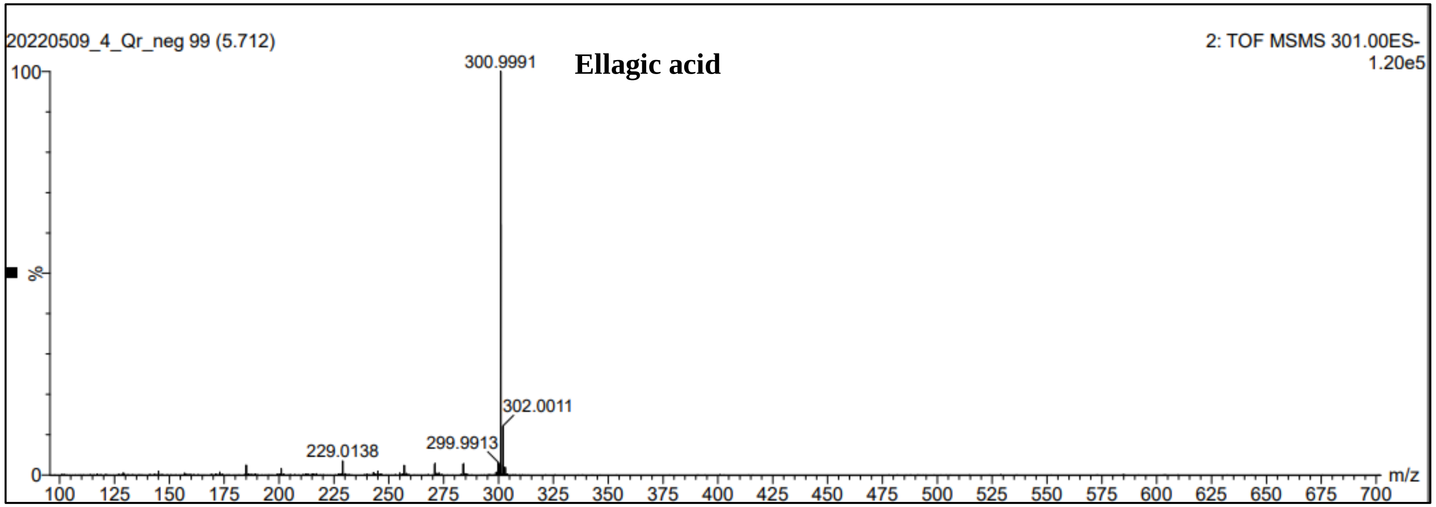

**Figure S6. TOF-MS/MS spectra of deprotonated compounds 17**

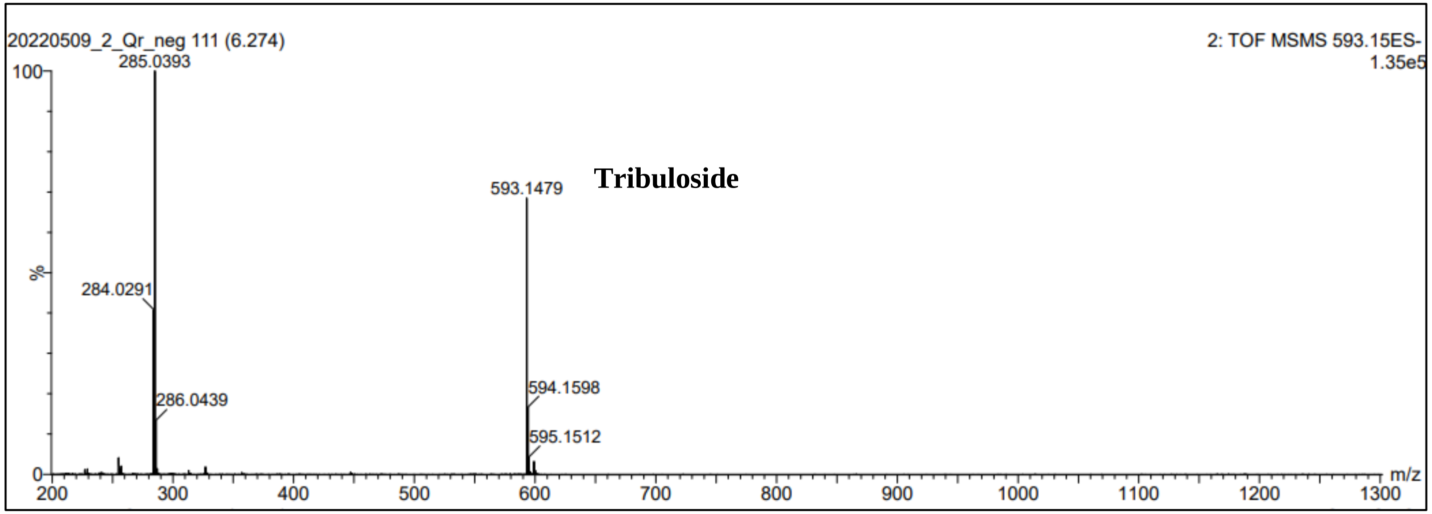


**Figure S7. TOF-MS/MS spectra of deprotonated compounds 18**

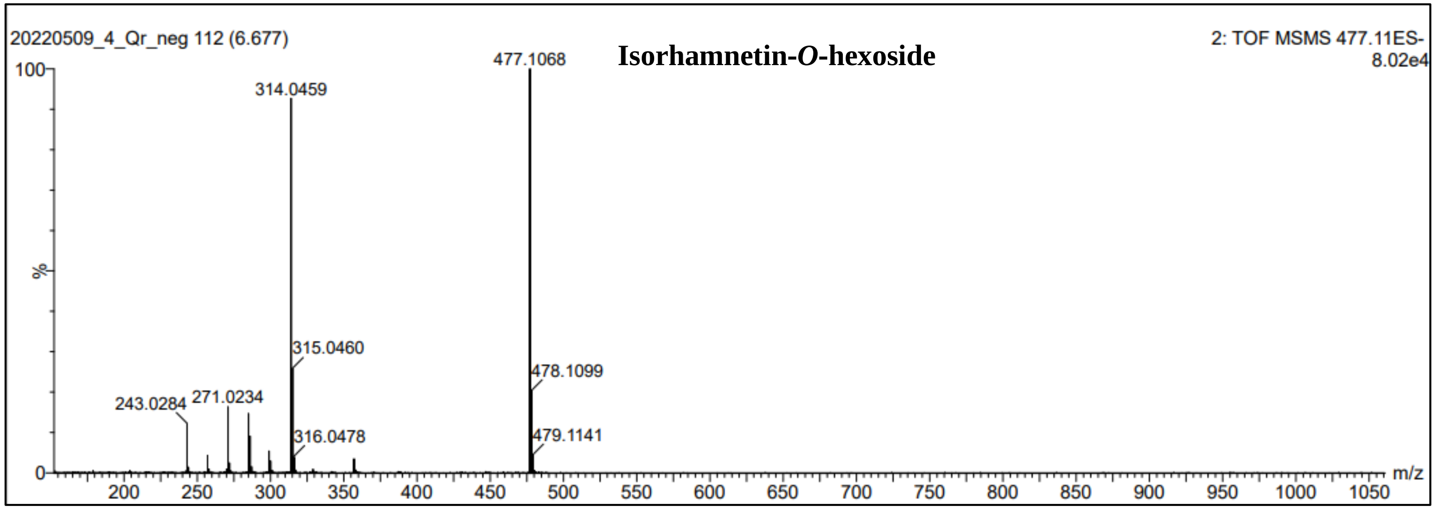

**Figure S8. TOF-MS/MS spectra of deprotonated compounds 22**

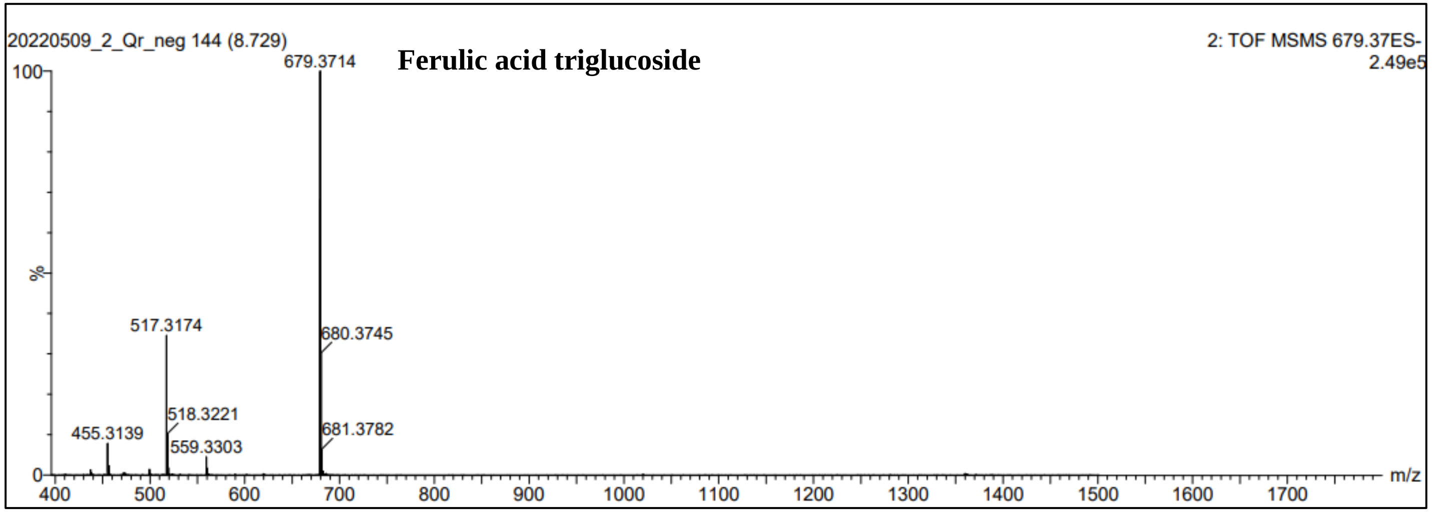


**Figure S9. TOF-MS/MS spectra of deprotonated compounds 25**

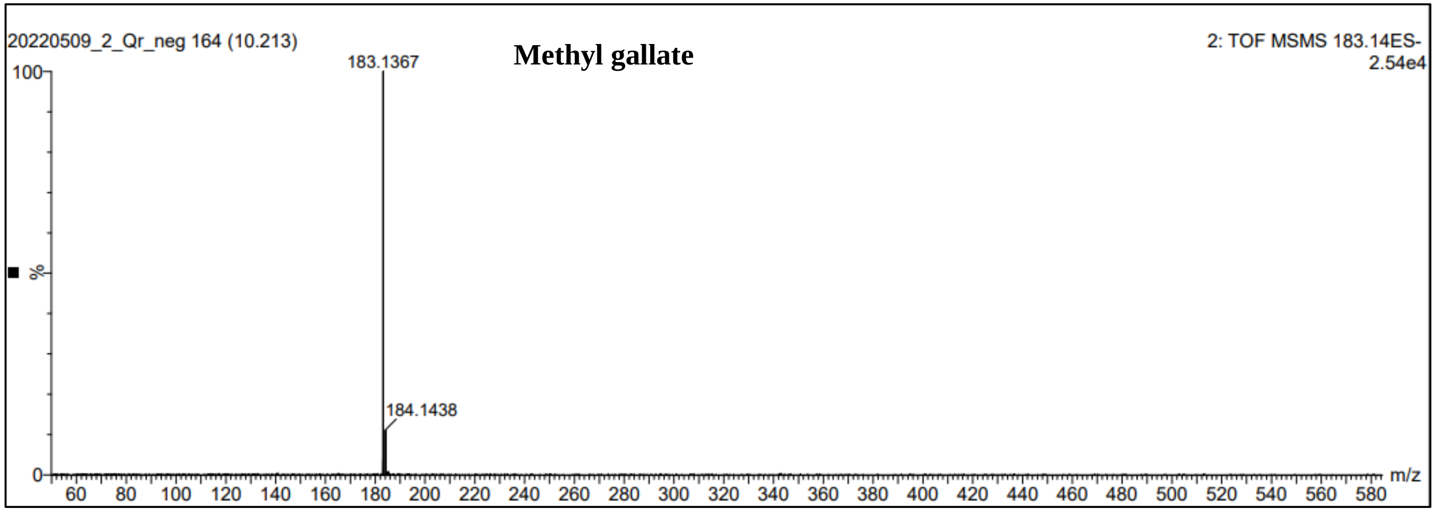

**Figure S10. TOF-MS/MS spectra of deprotonated compounds 27**

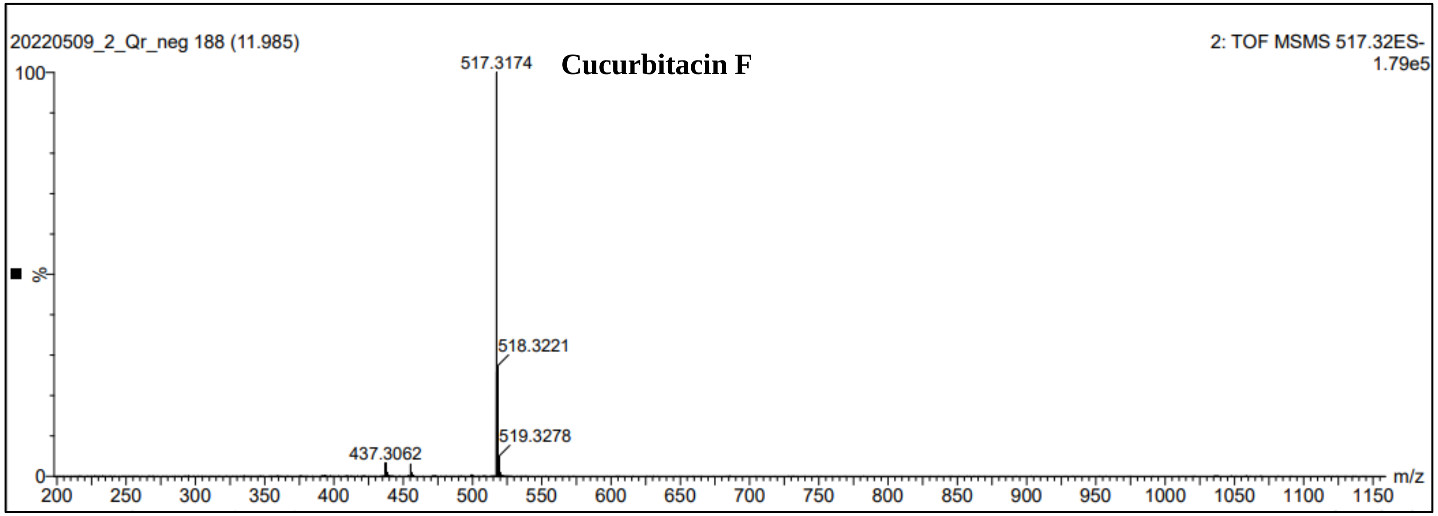


**Figure S11. TOF-MS/MS spectra of deprotonated compounds 28**
